# Spiradenocarcinoma: SEER Study of Epidemiology, Survival, and Treatment Options

**DOI:** 10.3390/jcm12052045

**Published:** 2023-03-04

**Authors:** Jérôme Martineau, Solange N. Walz, Matteo Scampa, Salvatore Giordano, Daniel F. Kalbermatten, Carlo M. Oranges

**Affiliations:** 1 Department of Plastic, Reconstructive, and Aesthetic Surgery, Geneva University Hospitals, Geneva University, 1205 Geneva, Switzerland; 2 Department of General and Plastic Surgery, Turku University Hospital, University of Turku, 20014 Turku, Finland

**Keywords:** epidemiology, survival, spiradenocarcinoma, treatment, SEER, spiradenoma, skin, malignant, eccrine, adnexal tumor

## Abstract

(1) Background: Spiradenocarcinoma is an extremely rare malignant adnexal tumor and there are only few studies on survival outcomes. Our aim was to perform an analysis of the demographic and pathological characteristics, treatment patterns, and survival outcomes of patients affected by spiradenocarcinoma. (2) Methods: The Surveillance, Epidemiology, and End Results program database of the National Cancer Institute was searched for all cases of spiradenocarcinoma diagnosed between 2000 and 2019. This database is considered representative of the US population. Demographic, pathological, and treatment variables were retrieved. Overall and disease-specific survival were computed according to the different variables. (3) Results: 90 cases of spiradenocarcinoma (47 females, 43 males) were identified. Mean age at diagnosis was 62.8 years. Regional and distant disease at diagnosis were rare, occurring in 2.2% and 3.3% of cases, respectively. Surgery alone was the most frequent treatment (87.8%), followed by a combination of surgery and radiotherapy (3.3%) and radiation therapy only (1.1%). Five-year overall survival was 76.2% and five-year disease-specific survival was 95.7%. (4) Conclusions: Spiradenocarcinoma equally affects males and females. Regional and distant invasion rates are low. Disease-specific mortality is low and is probably overestimated in the literature. Surgical excision remains the main form of treatment.

## 1. Introduction

Spiradenocarcinoma, or malignant eccrine spiradenoma (MES), is a very rare malignant cutaneous adnexal tumor, first described by Dabska et al. in 1971, that often arises from benign spiradenoma and less frequently develops de novo [1,2,3,4,5]. The delay in benign-to-malignant transformation is highly variable and has been reported to be around 20–30 years (ranging between 6 months to 70 years) [1,6]. Malignant transformation is characterized by the sudden growth of a stable chronic skin lesion, and associated with pain and ulceration of the tumor [1,2,3]. Given the rarity of this neoplasm and the evolution in imaging techniques, procedures used for diagnosis and initial staging are disparate. Histopathological analysis is required to confirm the diagnosis and can be difficult. Indeed, while high-grade carcinomas present an abrupt transition between benign spiradenoma and spiradenocarcinoma areas, lower-grade carcinomas can present a similar appearance and necessitate ancillary tests such as immunohistochemical staining for Ki-67, p53, and S100 [3,7,8]. MRI and FDG PET-CT are recommended imaging modalities for tumor staging [3,9,10]. Spiradenocarcinoma, especially if high-grade, is described as an aggressive tumor due to high rates of metastasis, local tumor recurrence rate above 20% following initial therapy—with a median time until tumor recurrence of 12 months—and high mortality [3,4,11,12]. However, most of the literature on spiradenocarcinoma stems from case reports, small case series, and reviews [2,3,4,9,13]. Due to the scarcity of cases, there are no treatment guidelines, although surgical excision has been advocated as the central pillar of treatment [3,4,6,9,14].

Considering that the head and neck region is one of the most often affected locations, surgical treatment can be challenging, especially in cases of large lesions. Indeed, due to aesthetic and functional concerns, excision and reconstruction must be carefully planned in close collaboration with dermatologists and plastic surgeons.

The aim of this study is to offer recent and reliable data on the epidemiological characteristics of spiradenocarcinoma and to analyze the different treatment modalities by using cases from the Surveillance, Epidemiology, and End Results (SEER) program of the National Cancer Institute, a database covering 26.5% of the US population and thus considered representative of the country’s demographics [15].

Studies on adnexal carcinomas based on data from the SEER database have been previously performed, but none focused solely on spiradenocarcinoma [16,17,18].

## 2. Materials and Methods

### 2.1. Patient Selection

The National Cancer Institute (NCI) Surveillance, Epidemiology, and End Results (SEER) database (https://seer.cancer.gov/) was searched across 17 different registries for all spiradenocarcinoma cases (8403/3 malignant eccrine spiradenoma) between 2000 and 2019. Data were extracted from the survival section of the SEER*Stat software (version 8.4.0.1). Ethical board approval was not necessary as the SEER program provides public domain data without personal medical identifiers.

### 2.2. Variable Selection

Selected variables included patients’ demographic information (sex, age at diagnosis, race, marital status, and the year of diagnosis), clinicopathological characteristics (staging, disease location, tumor size, histological grade, survival in months, vital status recode, and cause of death), and treatment patterns (surgery at primary site, type of surgery, radiotherapy, and chemotherapy).

The SEER database offers a standardized and simplified staging system derived from AJCC classification to ensure consistency in definitions over time. Localized cancer is generally defined by the disease being limited to the organ in which it started, without evidence of further spread. Regional cancer is determined by the disease having spread beyond its primary site to nearby lymph nodes or adjacent tissues, and distant cancer refers to the disease having spread away from the primary site to distant organs or lymph nodes. Finally, unstaged cancer describes cases without enough information to determine a stage. Different types of surgical excision are described, with local excision being without a margin, gross excision with a <1 cm margin, and wide excision with a >1 cm margin.

The “merging” tool offered by SEER*stat was used to create a variable combining different treatment patterns. Four subgroups were created: surgery with radiation, surgery without radiation, radiation therapy only, and no surgery and no radiation.

For a clearer interpretation, we subdivided patients by age at diagnosis into three categories: <50, 50–69, and ≥70. These three age categories were arbitrarily chosen to divide the cases around the median age at diagnosis for spiradenocarcinoma, which is considered to be around 60 years [3,11].

### 2.3. Statistical Analysis

Statistical analysis was performed using IBM SPSS version 28 (IBM, Armonk, NY, USA). The Kaplan–Meier method was used to determine overall survival (OS) and disease-specific survival (DSS). Univariable comparison in survival between subgroups was carried out using the log-rank test. Statistical significance was set at a *p*-value < 0.05.

## 3. Results

A total of 90 cases of spiradenocarcinoma diagnosed between 2000 and 2019 were retrieved. (Table 1) The incidence of cases increased linearly, with 18 cases (20%) diagnosed between 2000 and 2004, 21 cases (23.3%) between 2005 and 2009, 23 cases (25.6%) between 2010 and 2014, and 28 cases (31.1%) between 2015 and 2019. Average age at diagnosis was 62.8 years (SD 16.2), ranging between 25 and 99 years with a median age of 63 years (distribution: 22.2% <50 years, 43.4% 50–69 years, 34.4% ≥70 years). Females represented 52.2% of the cases and males 47.8%. The tumors affected mainly white patients (82.2%). The most common primary sites were the skin of the trunk (30%) and skin of the head and neck (30%), followed by the skin of the upper limb (16.7%) and skin of the lower limb (16.7%). There was one case in which the tumor affected the parotid gland. Histological grade was reported for only 17 cases out of 90 (18.9%), with 2 (2.2%) cases in which the tumor was well differentiated, 8 cases (8.9%) with a moderately differentiated tumor, and 7 (7.8%) cases with a poorly differentiated tumor. Localized disease was the most frequent stage at diagnosis (65.6%), while regional (3.3%) and distant (2.2%) disease were rare. Data describing tumor size were present in 50 cases, with a mean size of 37.6 mm and median size of 30 mm, ranging from 4 to 200 mm. In terms of treatment patterns, surgery alone was performed for 79 patients (87.8%), a combination of surgery and radiation therapy was performed for 3 patients (3.3%), and radiation therapy alone was performed in 1 case (1.1%). Eight patients did not undergo surgical treatment (8.9%); it was not recommended for 6 patients (6.7%) and was recommended but not performed for unknown reasons for 2 patients (2.2%). The most frequent type of surgery performed was a local tumor excision in 36 (40%) cases, followed by wide excision with margins >1 cm in 25 (27.8%) cases, and biopsy followed by gross excision in 15 (16.7%) cases.

**Figure 1:**
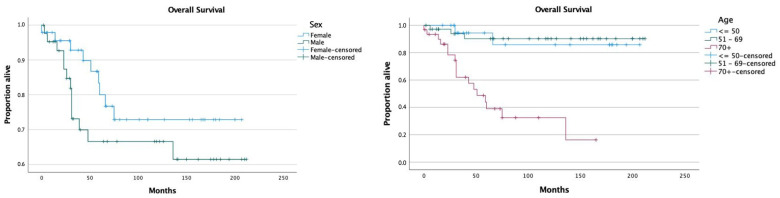
Survival distribution according to age and sex.

Mean overall survival (mOS) was 157 months (95% CI 137.7–176.3), with 96.6% alive at 1 year, 84.2% alive at 3 years, and 76.2% alive at 5 years. Five-year disease-specific survival (DSS) was 95.7%, with 4 out of 90 patients having a cause of death attributable to their spiradenocarcinoma diagnosis. There was no significant difference in mOS between females and males, with a mOS of 163.9 months (95% CI 139.5–188.3) and 146.3 months (95% CI 117.3–175.2) respectively (*p* = 0.218). Patients with an age below 50 years had a mOS of 183.3 months (95%CI = 152.6–214); mOS for patients between 50 and 69 was 194.5 months (95%CI = 175.6–213.4), and mOS was 73.9 months (95%CI = 50.2–97.7) among patients older than 70 years (Figure 1). OS of patients aged below 50 years was significantly higher than that of patients aged 70 years or more (log-rank, *p* < 0.05). Patients aged between 50 and 69 also had a significantly higher survival than patients older than 70 years at diagnosis (*p* < 0.05). There was no significant difference in terms of survival across the <50 years and the 50–69 years groups (Figure 1).

**Figure 2:**
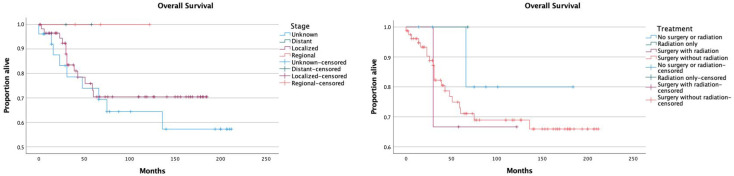
Survival distribution according to disease stage and treatment modalities.

OS did not differ significantly among ethnicities. Widowed patients had a significantly worse OS compared to married patients (*p* < 0.05), patients with an unknown marital status (*p* < 0.05), and divorced patients (*p* < 0.05); OS was similar across other marital status groups. Survival analyses based on histological grade were not performed because of the lack of available data. No significant differences in survival were observed across different stages. Surgery with radiation was associated with a lower OS compared to surgery without radiation (*p* < 0.05); OS was not significantly different between other treatment groups (Figure 2). OS was comparable between the different types of surgery.

## 4. Discussion

Spiradenocarcinoma is an extremely rare neoplasm with an estimated incidence of 0.07 cases per million person-years [19]. Due to the paucity of cases, very few studies are available. Most of the published data come from case reports and small case series. To our knowledge, this study is the largest and most comprehensive registry-based study on spiradenocarcinoma to date.

In line with recent reviews, our results show that spiradenocarcinoma is more frequent in the older population, with a median age at diagnosis of 63 years, with an even distribution between males and females and a predominance in white patients [3,9,17]. We found a linear increase in the incidence of cases over the years, which could be related to the aging of the US population, given the fact that it mostly affects older patients, and to the expansion of the population included in the SEER database over the years [20]. No propensity for a particular location was found, consistent with published reviews [3,9]. It is a tumor that almost exclusively affects the skin and, less frequently, genitalia—one patient in this study developed a spiradenocarcinoma of the parotid gland. A similar case of a spiradenoma of the parotid gland has been described in the literature, but these are extremely rare [21].

Unfortunately, in our cohort, tumor grade was reported in less than 20% of cases, which did not allow for analysis. Other studies have shown that higher-grade tumors are associated with a significantly worse prognosis and more aggressive behavior compared to low-grade spiradenocarcinoma [3,17,22].

Interestingly, only a few patients in our analysis presented with regional (3.3%) or distant disease (2.2%) at diagnosis, which is in stark contrast with previously published reports. In their meta-analysis, Andreoli et al. reported the presence of regional and distant disease in 16.7% and 33.3% of patients, respectively. Staiger et al. reported the presence of a distant metastatic spread in 19% of the cases, and Wagner et al. reported a metastasis occurrence rate of 37.4%, but did not report the rates of regional and distant spread separately [3,4,9]. We hypothesize that the lower rate in this cohort could partially be explained by the fact that 28.9% of the included cases were classified as unknown, but in view of the very high DSS rate in our study, a majority of these unstaged cases were probably localized at diagnosis. However, a more plausible explanation for this discrepancy is a probable selection bias since nearly all the data included in these reviews originated from case reports and case series, which are more likely to inform on complicated cases where there is a presence of advanced disease.

There is no consensus on the treatment of spiradenocarcinoma and the optimal treatment strategy is yet to be defined. Nevertheless, the mainstay of treatment is surgical resection of the tumor. Andreoli et al. reported a disease-free survival rate of 100% at 33-month follow-up in patients with localized spiradenocarcinoma that underwent surgical resection [4]. He recommended wide-margin resection given the aggressive course of the disease [4]. Other authors have also mentioned Mohs micrographic surgery (MMS) as a surgical option, highlighting its role in tumors that may present a subclinical extension and its aesthetic advantages in cosmetically sensitive zones such as the face [14,23,24,25]. The role of regional lymph node biopsy is currently unclear, with some authors arguing that it may be useful [4]. Conversely, the dissection of locoregional lymph nodes should not be routinely performed, as its benefits are unknown [3,26]. In patients with positive lymph nodes, lymph node excision is recommended [3,4,9,27].

Recommendations on adjuvant therapies are lacking and their role is yet to be determined. Data on radiotherapy in advanced disease is scarce and inconsistent, especially considering the fact that sweat gland tumors are known to be radioresistant [3,24,25,28]. The use of multiple chemotherapeutic agents for spiradenocarcinoma has been described over time without clear data on the optimal regimen. Based on one case report of a patient with metastatic disease who underwent successful treatment with tamoxifen, immunohistochemical analysis for estrogen receptors is recommended [27]. Recent case reports have evaluated the role of immunotherapy with PD-1 inhibitors in treatment of metastatic spiradenocarcinoma, with one case showing initial improvement but progression after 10 months and another showing a good response to immunotherapy. The authors suggest PD-L1 immunohistochemical analysis [6,29]. Further investigations are required regarding the role of adjuvant therapies in spiradenocarcinoma. In our cohort, 91.1% of patients underwent surgical treatment, 4.4% underwent radiation therapy, and none were treated with chemotherapy. Regarding clinical follow-up, Wagner et al. suggest reviews at 3-month intervals in the first year, at 6-month intervals in the second year, and annually after 2 years, and recommend regional lymph node examination, X-ray, and liver function tests [9].

The high DSS in the present study suggests that death from spiradenocarcinoma remained rare. However, no precise definitions of death from other causes were provided. This may have resulted in a limitation, as one could hypothesize that cause of death could be related to indirect repercussions of a tumor. Furthermore, the high DSS can be explained by the fact that spiradenocarcinoma generally affects older patients that are at higher risk of having multiple comorbidities and dying from causes other than the tumor. Overall survival was high, with 76.2% of patients alive at 5 years, in line with a 5-year OS rate of 76.7% reported by Avraham et al. [17]. These results indicate that spiradenocarcinoma could be less aggressive than is described in the literature, which is in accord with survival studies done on other rare adnexal carcinomas [17,30]. Specifically, in our survival analysis, we found that OS was significantly lower in patients older than 70 years. Widowed patients also exhibited poorer survival, which is a well-known association in cancer patients [31]. Patients who were treated with surgical resection with radiation therapy had a lower OS compared to patients who only underwent surgery. This is not a surprising finding, given that radiation therapy is used in cases with greater disease extension and hence more aggressive forms. We did not find significant survival differences between other variables. Recurrence rate was not available in the SEER database, rendering a disease-free survival (DFS) analysis unfeasible—a DFS analysis would be of particular importance in comparing surgical therapies. Such an analysis could provide evidence in favor of less aggressive tumor resections and, consequently, validate the use of less invasive surgical approaches that reduce cosmetic sequelae after resection.

The current population-based study was performed based on data collection and patient records that could have led to some bias. While demographic data were almost completely available, clinicopathological characteristics were often missing from the SEER database, and complete staging, grading information, and immunohistochemical staining data were lacking in most cases. The small sample size reduced the chance of findings reaching statistical significance, thus limiting analysis across different subgroups. Differences in OS between subgroups in this study should be interpreted cautiously owing to these confounding factors, especially given the low mortality rate and the limited availability of data for some variables.

## 5. Conclusions

Spiradenocarcinoma is a very rare disease that predominantly affects elderly white patients. This large and comprehensive registry-based study suggests that there may be an overestimation of regional or distant metastatic rates and that the disease could be less aggressive than is described in the current literature. Surgical treatment remains the standard of care and further investigations are required to assess the efficacy of adjuvant therapies in advanced disease. Additional studies with disease-free survival analysis are required to compare the recurrence rates associated with different surgical approaches—this could potentially support treatment with less invasive surgical therapies.

## Figures and Tables

**Table 1:** Demographic, clinicopathological, and treatment characteristics of spiradenocarcinoma patients.

	*n* (%)
Age at diagnosis	
*<50*	*20 (22.2)*
*50–69*	*39 (43.4)*
*≥70*	*31 (34.4)*
Sex	
*Male*	*43 (47.8)*
*Female*	*47 (52.2)*
Ethnicity	
*White*	*74 (82.2)*
*Black*	*4 (4.4)*
*Asian or Pacific Islander*	*7 (7.8)*
*Unknown*	*5 (5.6)*
Marital status	
*Married*	*35 (38.9)*
*Divorced*	*8 (8.9)*
*Separated*	*1 (1.1)*
*Single*	*17 (18.9)*
*Widowed*	*12 (13.3)*
*Unknown*	*17 (18.9)*
Year of diagnosis	
*2000–2004*	*18 (20)*
*2005–2009*	*21 (23.3)*
*2010–2014*	*23 (25.6)*
*2015–2019*	*28 (31.1)*
Primary site	
*C07.9—Parotid gland*	*1 (1.1)*
*C21.0—Anus, NOS*	*1 (1.1)*
*C44.1—Eyelid*	*1 (1.1)*
*C44.2—External ear*	*4 (4.4)*
*C44.3—Skin other/unspec. parts of face*	*10 (11.1)*
*C44.4—Skin of scalp and neck*	*12 (13.3)*
*C44.5—Skin of trunk*	*27 (30)*
*C44.6—Skin of upper limb and shoulder*	*15 (16.7)*
*C44.7—Skin of lower limb and hip*	*15 (16.7)*
*C44.9—Skin, NOS*	*1 (1.1)*
*C51.0—Labium majus*	*1 (1.1)*
*C51.9—Vulva, NOS*	*1 (1.1)*
*C60.9—Penis, NOS*	*1 (1.1)*
Grade	
*Well differentiated; Grade I*	*2 (2.2)*
*Moderately differentiated; Grade II*	*8 (8.9)*
*Poorly differentiated; Grade III*	*7 (7.8)*
*Unknown*	*73 (81.1)*
Stage	
*Localized*	*59 (65.6)*
*Regional*	*3 (3.3)*
*Distant*	*2 (2.2)*
*Unstaged*	*26 (28.9)*
Tumor size	*n = 49*
*Mean*	*37.6 mm*
*Std deviation*	*33.1 mm*
*Range*	*4–200 mm*
Treatment	
*No surgery or radiation*	*7 (7.8)*
*Radiation only*	*1 (1.1)*
*Surgery with radiation*	*3 (3.3)*
*Surgery without radiation*	*79 (87.8)*
Type of surgery	
*None*	*8 (8.9)*
*Local tumor excision*	*36 (40)*
*Biopsy of primary tumor followed by a gross excision*	*15 (16.7)*
*Mohs surgery*	*4 (4.4)*
*Wide excision or re-excision of lesion with margins* > *1 cm*	*25 (27.8)*
*Major amputation*	*1 (1.1)*
*Unknown*	*1 (1.1)*
Reason for no surgery	
*Not recommended*	*6 (6.7)*
*Recommended but not performed, unknown reason*	*2 (2.2)*
*Surgery performed*	*82 (91.1)*

## Data Availability

Not applicable.
